# Drug Interaction With Advanced Therapies in Inflammatory Bowel Diseases: A Blind Spot to Tackle

**DOI:** 10.1002/ueg2.12750

**Published:** 2025-01-06

**Authors:** Nathan Grellier, Julien Kirchgesner

**Affiliations:** ^1^ Department of Gastroenterology Saint‐Antoine Hospital, Assistance Publique‐Hôpitaux de Paris APHP Paris France; ^2^ INSERM Institut Pierre Louis d'Epidémiologie et de Santé Publique Sorbonne Université Paris France

**Keywords:** drug interaction, etrasimod, inflammatory bowel disease, sphingosine‐1‐phosphate receptor modulators, ulcerative colitis

Polypharmacy is common in patients with inflammatory bowel diseases (IBD) due to the condition's complexity and associated comorbidities. With the increasing number of advanced therapies, the risk of drug‐drug interactions increases particularly in the elderly, potentially compromising treatment efficacy and safety. Serious adverse effects such as severe leukopenia with methotrexate and cotrimoxazole highlight this risk. However, monoclonal antibodies generally exhibit safer drug interaction profile compared to small molecules, while data on drug interactions with small molecules such as JAK inhibitors and sphingosine‐1‐phosphate (S1P) receptor modulators remain limited.

S1P is a membrane‐derived lysophospholipid signaling molecule involved in various physiological processes such as regulation of the immune system, nervous system, and cardiovascular metabolism [1]. Ozanimod and etrasimod are two S1P receptor modulators approved for the treatment of moderate‐to‐severe ulcerative colitis (UC) in both induction and maintenance settings [2, 3]. Several class‐specific adverse events (AEs) have been reported in the Phase 3 trials, including macular edema, hypertensive crisis, and hypertension. These AEs have been reported with low incidence, but potentially serious complications may occur, particularly blindness and cardiovascular events. In addition, potential drug‐drug interactions have been hypothesized due to the molecular structure of ozanimod. In vitro pharmaceutical studies have shown that ozanimod may interact with monoamine oxidase B and increase tyramine levels, which may provide a mechanistic explanation for hypertensive crisis [4]. No such interaction has been described with etrasimod, which is metabolized by several types of cytochromes [5]. Although there is no clinical evidence to corroborate these data, several questions remain regarding the potential interaction between S1P modulators and inhibitors of monoamine oxidase (IMAO) such as antidepressants and opioids. Regarding drug interactions, up to 20% of patients with UC use antidepressants after UC diagnosis and one third use opioids after hospital discharge [6, 7]. Due to their serotoninergic side effects, there is a potential risk of drug interaction with S1P modulators, especially serotonin syndrome.

In the latest issue of the UEG Journal, Afzali and colleagues assessed the safety of using etrasimod alongside opioids or antidepressants through a post‐hoc analysis of the Phase 3 ELEVATE UC 12 and ELEVATE UC 52 trials [8]. They aimed to assess the proportion of patients with potential serotoninergic AEs, defined as neuroleptic malignant syndrome, serotonin syndrome (pyrexia and tachycardia), Hunter criteria (myoclonus, agitation, tremor, hyperreflexia, hypertonicity, diaphoresis), or hypertension‐related AEs. Among 527 patients with moderate‐to‐severe UC receiving etrasimod, 14.6% used opioids (mostly fentanyl) and 6.6% used antidepressants, including selective serotonin reuptake inhibitors (SSRIs), serotonin‐norepinephrine reuptake inhibitors (SNRIs), tricyclic antidepressants, and MAO inhibitors. Patients were divided into four subgroups: etrasimod with or without opioids, and with or without antidepressants. The rate of serotoninergic AEs was similar across groups. Pyrexia occurred in 2.6% of patients using opioids versus 4.0% without opioids and in 2.6% of those using antidepressants versus 3.9% without antidepressants. Tachycardia and hypertension were rare, with only two hypertension cases in the antidepressant group and none in the opioid group. Agitation occurred in one patient, and no AEs were severe or led to etrasimod discontinuation. Despite substantial use of alcohol and tobacco (20%–40%) in the study population, no increased risk of serotoninergic AEs was observed with opioids or antidepressants. These findings strengthen the safety profile of etrasimod, suggesting no significant drug‐drug interactions with these medications.

Some limitations need to be acknowledged. This is a post‐hoc analysis of clinical trials including a small number of patients with uncertain exposure of opioids and antidepressants during follow‐up, but the low number of events and the absence of excess risk in patients exposed to other substances strengthen the validity of this study. Recently, Regueiro and colleagues reported similar results from ozanimod Phase 3 trials using the same methodology [9]. These data highlight the relative safety of S1P modulators regarding drug interactions, though bradycardia and macular edema remain key considerations. Pre‐treatment electrocardiograms and ophthalmoscopy in patients at increased risk of macular edema such as diabetic patients, are advised to mitigate these risks. While cases of progressive multifocal leukoencephalopathy (PML) have been reported with S1P modulators in multiple sclerosis, no PML warnings have been reported for now in IBD patients treated with S1P modulators [10].

Finally, etrasimod appears to be a safe treatment for moderate to severe UC without serotonergic effects when co‐prescribed with antidepressants or opioids. However, as the IBD population age and multimorbidity increase, the potential for drug interactions with S1P modulators highlights a growing topic that will require more research in the era of small molecules.

## Conflicts of Interest

The authors declare no conflicts of interest.

## Data Availability

The authors have nothing to report.
